# Construction of a Codon-Adapted Nourseotricin-Resistance Marker Gene for Efficient Targeted Gene Deletion in the Mycophenolic Acid Producer *Penicillium brevicompactum*

**DOI:** 10.3390/jof5040096

**Published:** 2019-10-10

**Authors:** Yasaman Mahmoudjanlou, Birgit Hoff, Ulrich Kück

**Affiliations:** Allgemeine & Molekulare Botanik, Ruhr-Universität Bochum, 44780 Bochum, Germany; yasaman.mahmoudjanlou@rub.de (Y.M.); birgit.hoff@basf.com (B.H.)

**Keywords:** *Penicillium brevicompactum*, codon-adapted nourseothricin resistance gene, homologous recombination, *MAT1-2-1*, *flbA*

## Abstract

*Penicillium brevicompactum* is a filamentous ascomycete used in the pharmaceutical industry to produce mycophenolic acid, an immunosuppressant agent. To extend options for genetic engineering of this fungus, we have tested two resistance markers that have not previously been applied to *P. brevicompactum*. Although a generally available phleomycin resistance marker (*ble*) was successfully used in DNA-mediated transformation experiments, we were not able to use a commonly applicable nourseothricin resistance cassette (*nat1*). To circumvent this failure, we constructed a new *nat* gene, considering the codon bias for *P. brevicompactum*. We then used this modified *nat* gene in subsequent transformation experiments for the targeted disruption of two nuclear genes, *MAT1-2-1* and *flbA*. For *MAT1-2-1*, we obtained deletion strains with a frequency of about 10%. In the case of *flbA*, the frequency was about 4%, and this disruption strain also showed reduced conidiospore formation. To confirm the deletion, we used *ble* to reintroduce the wild-type genes. This step restored the wild-type phenotype in the *flbA* deletion strain, which had a sporulation defect. The successful transformation system described here substantially extends options for genetically manipulating the biotechnologically relevant fungus *P. brevicompactum.*

## 1. Introduction

Before the advent of DNA-mediated transformation systems in filamentous fungi, random mutagenesis and strain screening programs were the only genetic approaches to strain improvement in biotechnologically relevant fungi. However, these mutagenesis methods often resulted in strains that had deleterious effects on fungal growth, sporulation, and genome stability. In the past 40 years or so, different directed transformation systems have been established to overcome these problems [1]. Such systems deciphered diverse gene functions, based on targeted gene disruption or overexpression experiments [2]. Previously, advanced transformation strategies were developed for filamentous fungi, such as CaCl_2_/polyethylene glycol, electroporation, particle bombardment, and *Agrobacterium tumefaciens*–mediated transformation [3,4,5]. Moreover, RNA interference and CRISPR/Cas9 systems have been described as suitable approaches to genetic manipulation of fungi [6,7,8]. 

An efficient transformation strategy requires a suitable screening system and effective selectable markers. Antibiotic resistance genes of bacterial origin are the most frequently used markers in filamentous fungi because no mutant recipient strains are needed to select transformants. In contrast, auxotrophic marker systems require construction of appropriate recipients. The hygromycin B resistance (*hph*) gene is an applicable selection marker for most fungi. However, because some *Aspergillus* species are relatively resistant to this antibiotic, the phleomycin resistance gene (*ble*) has been described as an alternative dominant selectable marker for these species [2,9]. Application of antibiotics neomycin, carboxin, fludioxonil, and blasticidin for the selection of transformants also has been suggested for genetic engineering of filamentous fungi [3,5,10].

Here, we have tested different bacterial resistance markers for the DNA-mediated transformation of the filamentous fungus *Penicillium brevicompactum*. In 1893, this filamentous ascomycete was discovered to be the first-known producer with antibacterial activity [11]. Since then, diverse *P. brevicompactum* strains have been described as producing a large range of bioactive compounds. These products, including secondary metabolites, are of significant biotechnological and medical importance because of their antiviral, antifungal, antibacterial, antitumor, and antipsoriasis activities [12]. Currently, the pharmaceutical industry uses *P. brevicompactum* for the large-scale biosynthesis of mycophenolic acid, an immunosuppressant agent with derivatives that are applied for autoimmune conditions as well as for kidney, heart, and liver transplantation patients [13].

Because of the limited number of selection markers, few studies have demonstrated genetic engineering of *P. brevicompactum.* In one report, the nitrate reductase gene from *Fusarium oxysporum* was used as a selection maker in an appropriate nitrate reductase-deficient recipient [14]. Successful *A. tumefaciens*–mediated transformation of *P. brevicompactum* has been described, as well, in which *hph* was used as a selectable marker under the control of the *gpdA* promoter from *Aspergillus nidulans* [15]. Here, we used *ble* and the nourseothricin-resistance marker *nat1* for gene deletion analysis, followed by complementation studies. Although the previously applied *ble* [16,17] worked properly in the complementation studies, the commercially available *nat1* could not successfully be used for selection of antibiotic-resistant *P. brevicompactum* transformants, although other studies have applied this resistance marker successfully in filamentous fungi [18,19]. To circumvent this difficulty, we developed a novel codon-adapted *nat1* selection marker that is suitable for high-frequency transformation of *P. brevicompactum*. Additionally, we demonstrated that this newly constructed gene together with *ble* is suitable for site-specific gene deletion experiments. Our findings extend the options for genetic manipulation of this important biotechnological fungus, which is the subject of metabolic engineering experiments, to optimize the production of mycophenolic acid and its analogues [13].

## 2. Material and Methods

### 2.1. Strains, Plasmids and Culture Conditions

All wild type and recombinant strains from *Penicillium brevicompactum* used in this investigation are shown in Table 1. The strains Dutch Centraalbureau voor Schimmelcultures CBS 257.29 and CBS 110068 were chosen for transformation and isolation of genomic DNA. To prepare the spore suspension, all *P. brevicompactum* strains were grown on Czapek Yeast Agar (CYA) [20] for 7 days at 27 °C. Medium M334, containing 2% glucose monohydrate, 2% tryptone, 0.3% potassium dihydrogen phosphate and 0.1% magnesium sulphate heptahydrate, was used as a minimal liquid growth medium. For sensitivity tests against antibiotics, we used a complete culture medium (CCM) [21].

For plasmid construction we used *Escherichia coli (E. coli*), K12 XL1-Blue. A standard cloning protocol was carried out for electroporation of *E. coli* cells [22].

### 2.2. Construction of Transformation Vectors

The custom-synthetized *Pbnat1* gene is codon-adapted and carries restriction sites for *Nco*I and *BamH*I that flank the gene. Prior to ligation, both *Pbnat1* and the vector pPtrpCnat1 were digested with *Nco*I and *BamH*I. The resulting plasmid, pPtrpC-Pbnat1, served for the construction of deletion vectors. To ensure high homologous recombination efficiency, we used about 1 kb of the 5’ and 3’ flanking regions of *MAT1-2-1* and *flbA*. The *MAT1-2-1*- 5’ flanking region was amplified by PCR using the oligonucleotides 5´-Pb-MAT1-2-1-MluI-for and 5’-Pb-MAT1-2-1-EcoRI-rv. Restriction with corresponding enzymes and N-terminal fusion to PtrpC-Pbnat1 were followed by C-terminal fusion of the 3’ flanking regions, which had been amplified by PCR using oligonucleotides 3’-Pb-MAT1-2-1-NotI-for/ 3’-Pb-MAT1-2-1-NotI-rv. For ligation, the amplicon was digested with *Not*I. We used the same strategy for the construction of the plasmid pPb-flbA-KO. The downstream fragment was generated by PCR using 5’-Pb-flbA-PstI-for/ 5´-Pb-flbA-PstI-rv as primers, restricted with *Pst*I, and fused N terminally to PtrpC-Pbnat vector. It then was amplified by PCR with primer pairs 3’-PbflbA-NotI-for/ 3’-flbA-NotI-rv and ligated into the *Not*I restriction site, which terminates the 3´end of *Pbnat1*. 

For complementation of the corresponding deletion strains, *MAT1-2-1* was amplified from genomic DNA of CBS 110068 using oligonucleotides MAT1-2-1-OE-EcoRI-for/ MAT1-2-1_EcoRI-OE-rv. To amplify *flbA*, we used as template genomic DNA from CBS 257.29, using oligonucleotides Pb-flbA-BglII-OE-for/ Pb-flbA-BamHI-OE-rv. Both genes were fused to the 3´-end of *Pgpd-egfp* and subsequently introduced separately into pN-EGFP [23]. *nat1* in pN-EGFP was further substituted by *ble*. The sequences of all oligonucleotides are given in Table S3, and plasmids in Table S4.

### 2.3. Bioinformatics and Programs

We obtained the genome sequences of AgRF18 strains from online database Joint Genome Institute (JGI) (https://genome.jgi.doe.gov/portal/). The JGI local blast tool was used to receive the sequences of *MAT1-2-1* and *flbA* genes. To generate the codon usage table, we used the Bioperl based fascodon program. The sequence of the synthetic *Pbnat1* gene was designed, using the online tool GENEius-The Tuning Tool (https://www.eurofinsgenomics.eu/en/gene-synthesis-molecular-biology/geneius/). Custom gene synthesis was done by GenScript Corporation, 120 Centennial Ave., Piscataway, NJ 08854, USA (https://www.genscript.com/). For calculation of CAI, we used the online CAIcal algorithm local version 1.3 (http://genomes.urv.es/CAIcal/) [24]. For in silico cloning strategies, we used SnapGene version 5.0, GSL Biotech LLC program (https://www.snapgene.com/). 

### 2.4. Transformation of P. brevicompactum Strains

DNA-mediated transformation of *P. brevicompactum* strains was performed with some slight modifications, as previously described by Bull et al. (1988) [25]. Briefly, strains were grown in M344 shaking liquid medium (230 rpm) at 25 °C for 24 h. Three grams of filtered mycelia were used for protoplast preparation. Protoplast preparation involved shaking the fungal mycelia at 110 rpm and 25 °C for 2h in 35 mL 0.9 M NaCl buffer containing 40 mg/mL Vinotaste®Pro enzyme. A total of 1 × 10^7^ protoplasts in 100 µL transformation buffer (0.9M NaCl + 0.9M CaCl_2_) was transformed with 10 µg linear or circular DNA by means of a second transformation buffer (50% PEG 6000, 50 mM CaCl_2_, 10 mM Tris; pH 5.0). For selection of transformants on solid complete culture medium supplemented with 2% glucose, we used 200 µg/mL nourseothricin or 100 µg/mL phleomycin. To obtain purified transformants from single colonies, we streaked spores on CCM medium containing selection antibiotics. Molecular analysis of transgenic fungal strains by PCR and Southern blotting and hybridization was done as described previously [19,22].

## 3. Results and Discussion

The aim of this study was to develop an alternative transformation system for targeted gene disruption in different strains of the biotechnologically relevant fungus *P. brevicompactum.*

### 3.1. Test for Sensitivity Against Antibiotics and DNA Transformation

**Figure 1:**
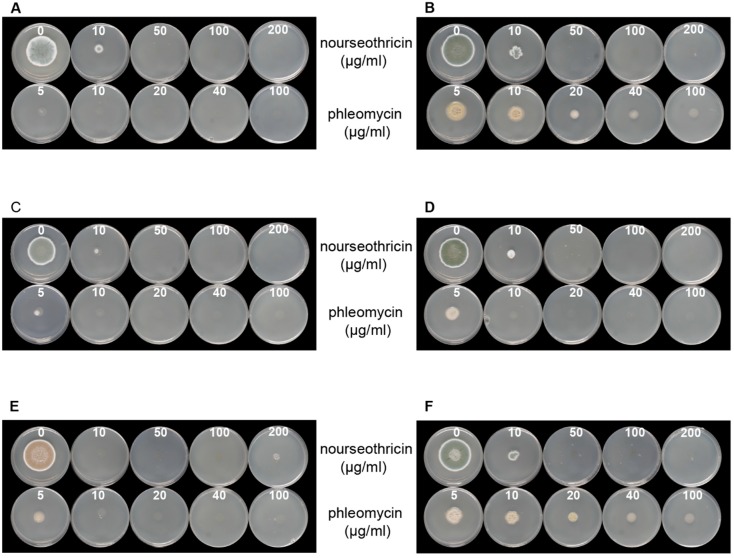
Test for sensitivity against different concentrations of antibiotics as given for six *P. brevicompactum* wild type strains. (**A**) CBS 257.29; (**B**) CBS 110068; (**C**) CBS 110070; (**D**) CBS110071; (**E**) CBS 317.59; (**F**) IBT 27083. Antibiotic concentrations (µg/mL) are indicated in white on each plate. For further transformation experiments, CBS 257.29 and CBS 110068 were selected.

To use bacterial resistance markers for DNA-mediated transformation, we tested the sensitivity of six type culture collection strains from *P. brevicompactum* against different concentrations of nourseothricin and phleomycin. Our results indicated that all wild-type strains (Table 1) are sensitive to even low concentrations of nourseothricin (Figure 1). Indeed, we found a rather high sensitivity in all strains tested, and even a low antibiotic concentration of 10 µg/mL resulted in growth reduction. This exposure is much lower than the 150 µg/mL previously used to select transformants of the related *P. chrysogenum* [26]. However, a higher phleomycin concentration is required for growth inhibition of CBS 110068 and IBT 23078. Thus, a concentration of 100 µg/mL is necessary for reliable selection of transformants on phleomycin-containing medium. 

To examine the suitability of *ble* as well as *nat1* for *P. brevicompactum* transformation, we used two transformation vectors that previously had been successfully applied for *Penicillium chrysogenum*: p1783-1 and pDrive/PtrpC-Tn5Phleo [21,27]. For hosts, we applied two strains carrying *MAT1-1-1* (CBS 257.29) or *MAT1-2-1* (CBS 110068). Both show a high production of 3.1 and 1.8 g mycophenolic acid per kilogram of mycelia, respectively, and thus can be considered as high producers of this immunosuppressant [28].

Using the vector pDrive/PtrpC-Tn5Phleo for transformation, we found a rather good frequency of transformants (about 6 per 10 µg DNA) for both recipient strains on phleomycin-containing plates. In contrast, the *nat1* gene (p1783-1) yielded no transformants in 15 individual transformation experiments. This gene originates from the prokaryote *Streptomyces nourseii*, and its codon usage is not adapted to the expression machinery of eukaryotic host systems. To optimize expression of *nat1*, we decided to synthesize it with a codon bias adapted to *P. brevicompactum*. 

### 3.2. Construction of a Codon-Adapted nat1 Gene

The successful expression of codon-optimized genes in diverse hosts from filamentous fungi has previously been reported. For example, a completely synthetized codon-optimized *flp* recombinase gene from yeast *Saccharomyces cerevisiae* allowed for successful establishment of a marker recycling system in *P. chrysogenum* and *Sordaria macrospora* [19]. Moreover, construction of a codon-adapted luciferase gene for expression in *Neurospora crassa* provided a novel reporter assay system for this fungus [29]. Furthermore, the efficient expression of a codon-optimized *Derf7* gene encoding a mite allergen in *Aspergillus oryzae* suggested that fungi can serve as hosts for the synthesis of recombinant allergen used in immunotherapy [30].

To improve the expression efficiency of *nat1* in *P. brevicompactum*, we determined the codon bias for the fungus. For this purpose, we accessed the available genome sequences of the *P. brevicompactum* strain AgRF18 (online database of the Joint Genome Institute: https://genome.jgi.doe.gov/portal/) to generate a codon usage table (Supplementary Table S1). Table 2 gives the corresponding frequencies of codon usages. We selected 12,343 protein-coding sequences (CDS) and 5,661,200 codons from *P. brevicompactum* and compared them with the codon usage of native *nat1* from *S. noursei*. This comparison yielded significant differences between the codon biases. For instance, AGT is the preferential codon for the amino acid aspartate in *P. brevicompactum* (51.8%) but is used at a frequency of only 4.4% in the native *nat1* gene, which shows a bias toward using AGC to encode aspartate (Table 2). Moreover, the GC content is quite different between *nat1* (71.2%) and the *P. brevicompactum* genome (52.8%). Because of these differences, we designed a novel *nat1* gene with a codon bias adapted to the codon-preferred bias of *P. brevicompactum*. 

**Figure 2:**
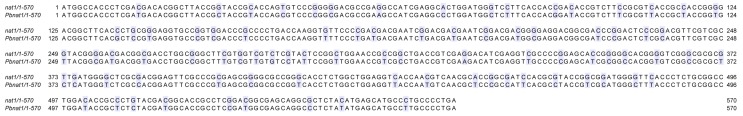
Comparative sequence alignment of codon adapted *Pbnat1* gene with the commercially available *nat1* gene. 82 nucleotide changes are marked in light blue.

To obtain the most suitable codon, we generated the codon-optimized gene in silico, as described in the materials and methods section. This modified sequence is given in Figure 2, and served for the customized gene synthesis. We designated the corresponding gene as *Pbnat1.* This novel *Pbnat1* gene with 88.4% DNA homology to *nat1* has a GC content of 63.7%, which is more similar to the overall GC content of the *P. brevicompactum* genome (52.8%). This modification is important because GC content is a main mediator of codon and amino acid usage and thus the most significant factor determining codon bias [31]. In total, in *Pbnat1*, we changed 7.5% of the first (GC1) and 23.2% of the third (GC3) codon positions (Supplementary Table S2).

To examine the compatibility *Pbnat1* with *P. brevicompactum* codon bias, we calculated the codon adaptation index (CAI) for both *nat1* and *Pbnat1*. For this purpose, we used the online CAIcal algorithm (http://genomes.urv.es/CAIcal/) [24]. The CAI for a specific gene is defined by comparing its codon usage rate and frequency in a reference set of genes. Assigned values are between 0 and 1. With CAI values closer to 1, the expression level of the targeted gene is expected to be better in a heterologous host system [19,32,33]. Changing about 43% of all codons shifted the CAI value for *Pbnat1* from 0.83 to 0.91. This result led us to predict that *Pbnat1* compared with *nat1* would have an optimized and more accurate expression in *P. brevicompactum.*

### 3.3. Use of the Codon-Optimized Pbnat1 Gene for Site-Specific Deletion of Two Nuclear Genes

To improve the transformation efficiency in *P. brevicompactum*, we developed a reliable procedure based on the use of protoplasts. For this purpose, we tested two buffers: potassium phosphate [14] and 0.9 M sodium chloride [25] for CBS 257.29 and CBS 110068. Based on our results, we concluded that 0.9 M sodium chloride containing 40 mg/mL Vinotaste®Pro digestive enzyme was the only effective buffer for protoplasting of *P. brevicompactum* strains. 

After establishing the transformation method (see the material and methods section), we investigated the efficiency of the newly constructed *Pbnat1* gene in *P. brevicompactum* using the vector pPtrpC-Pbnat1. This plasmid contains the *Pbnat1* gene under the control of the *trpC* promoter from *A. nidulans*. Application of *Pbnat1* led to successful transformation events at a frequency of about 10 nourseothricin-resistant transformants per 10 µg of circular DNA. All transformants were genetically stable on selective media and were propagated for more than 12 months. 

**Figure 3:**
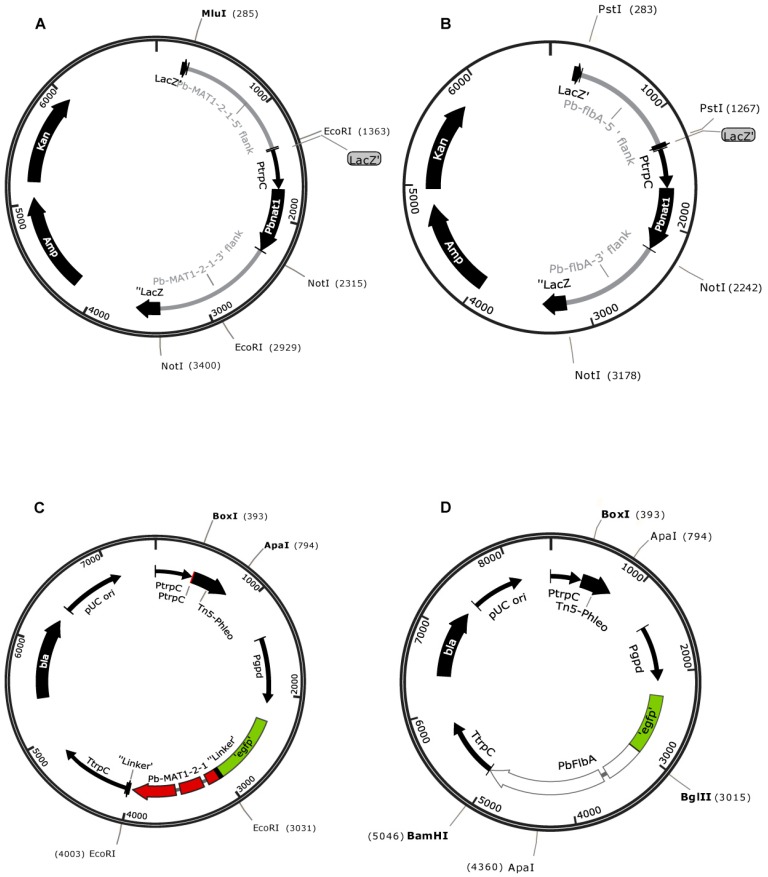
Maps of vectors for DNA mediated transformation of *P. brevicompactum*. (**A**,**B**) Plasmids pPb-MAT1-2-1-KO (**A**) and pPb-flbA-KO (**B**) for site-specific deletion of *MAT1-2-1* and *flbA* genes, carrying the codon adapted *pbnat1* gene under the transcriptional control of the *PtrpC* promoter; (**C**,**D**) Plasmids pPb-MAT1-2-1-comp (**C**) and pPb-flbA-comp (**D**) for complementation of the corresponding deletion strains. Both plasmids carry the *egfp*-tagged gene of interest under the constitutive *gpd* promoter from *A. nidulans.*

In the next step, we applied the *Pbnat1* gene containing the vector pPtrpC-Pbnat1 for site-specific deletion of two genes inferred to be involved in fungal development: *flbA* and *MAT1‐2‐1*. *flbA* encodes a regulator of G-protein signaling that is involved in asexual sporulation and mycelial proliferation, and *MAT1-2-1* encodes a transcription factor that controls sexual development [17,34,35]. To generate deletion mutants by homologous recombination, we transformed the strains with a linear deletion cassette containing 5‘-and 3’- flanking regions derived from the target gene and each about 1 kb long (Figures S1A and S2A). The corresponding cassettes were obtained from plasmids, shown in Figure 3A,B. CBS 257.29 and CBS 110068 served as the recipient strains for disruption of *flbA* and *MAT1-2-1,* respectively. With linear DNA for deletion of *MAT1-2-1*, we observed a frequency of 10–12 transformants per 10 µg of linear DNA. The transformation frequency was considerably less when we used the *flbA* deletion cassette (pPb-flbA-KO), with about 2–4 transformants per 10 µg of DNA. 

**Figure 4:**
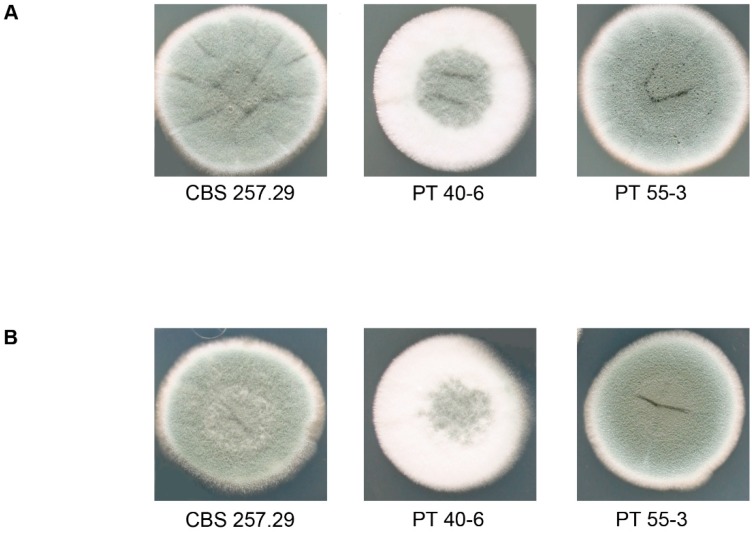
Phenotypic analysis of *flbA* deletion and corresponding complementation strains after 7 days of growth on CCM medium at 27 °C, under different light condition. (**A**) light, (**B**) dark. CBS 257.29: wild type; PT 40-6: deletion strain; PT 55-3: complementation strain.

To verify that homologous recombination had occurred, we performed PCR amplification and Southern hybridizations with a probe that was homologous to the 5’-flanking region of the target gene (Supplementary Figures S1B, S1C, S2B, S3F). Our data confirmed the presence of homokaryotic deletion strains. While the *MAT1-2-1* deletion strains showed no detectable phenotype, the *flbA* deletion strains were distinct from the wild type because of a delayed and reduced sporulation phenotype (Figure 4). This developmental feature is similar to previously reported phenotypes that resulted when the homologous *flbA* gene was deleted in different *Aspergillus* species [36,37]. Verification of 60 putative *MAT1-2-1* strains revealed a frequency of 10% for deletion of *MAT1-2-1* gene. In contrast, only 1 of 24 putative *flbA* deletion strains was identified as a homokaryotic deletion strain. 

In the next step, we conducted complementation experiments to confirm the successful deletion of the target genes. For complementation, we used the plasmids pPb-MAT1-2-1-comp and pPb-flbA-comp to transform the appropriate deletion strains (Figure 3C, 3D). PCR analysis and Southern hybridization with a target gene–specific probe served as confirmation of the successful ectopic integration of the wild-type genes (Figure S3). The ectopic integration of *flbA* into the deletion strains activated a wild type–like sporulation phenotype (Figure 4).

In our experiments we observed a sufficiently high frequency of homologous recombination in *P. brevicompactum*. Therefore, it is not necessary to use mutants in which non-homologous end joining (NHEJ) is abolished by mutation of the NHEJ repair system. *Neurospora crassa* was the first filamentous ascomycete in which homologous recombination was enhanced by disrupting genes for the catalytic subunit (DNA-PKcs) or the regulatory DNA-binding subunits (Ku70/80 heterodimer) [38,39]. Later, strains from diverse ascomycetes were generated to serve as recipients for the targeted integration of foreign genes [6,40]. However, several reports showed that non-homologous end joining–deficient strains accumulate random mutations and thus are less suitable for long-term experiments. Furthermore, these recipient strains show an elevated sensitivity to different chemicals such as bleomycin, methyl methanesulfonate, and ethyl methanesulfonate (for review, see [6,40]). Thus, some investigators have avoided using specific recipients for homologous recombination because the natural homologous recombination frequency was similar to that described here [41,42,43]. 

In conclusion, we have extended the molecular tools for genetic manipulation of the biotechnologically relevant fungus *P. brevicompactum*. The opportunity to generate deletion and complementation strains by the use of different marker genes opens up future avenues to research to identify factors that control or regulate secondary metabolism in this mycophenolic acid–producing fungus.

## Figures and Tables

**Table 1:** Fungal strains used in this investigation.

Strain	Characteristics, Genotype	Source
CBS 257.29	Wild type, neotype of *P. brevicompactum* Dierckx	(1)
CBS 317.59	Wild type	(1)
CBS 110068	Wild type	(1)
CBS 110070	Wild type	(1)
CBS 110071	Wild type	(1)
IBT 23078	Wild type	(2) [13]
∆PbMAT1-2-1	*MAT1-2-1Δ*::*ptrpC*::*Pbnat1*; recipient: CBS 110068	This study
∆PbflbA	*flbA*Δ::p*trpC*::*Pbnat1*; recipient: CBS 257.29	This study
∆PbMAT1-2-1::PbMAT1-2-1	*pgpd*::*egfp*::*PbMAT1-2-1:ptrpC*::*phle*	This study
∆PbflbA::PbflbA	*pgpd*::*egfp*::*PbflbA:ptrpC*::*phle*	This study

1. CBS-KNAW Collections—Westerdijk, Fungal Biodiversity Institute, Utrecht, Netherlands. 2. IBT, Culture Collection of Fungi, Mycology Group, BioCentrum-DTU, Technical University of Denmark, Lyngby, Denmark.

**Table 2:** Comparison of the codon usage of *nat1* and *Pbnat1* with the genomic codon usage of *P. brevicompactum* (*Pb*) (AgRF18), (https://genome.jgi.doe.gov/portal/). Numbers give the usage bias of each codon for each amino acid in percent. Preferred amino acid codons are labelled in red. First column in the left indicate the first base of triplets, upper row the second base and last column the third base.

	T				C				A				G				
		*nat1*	*Pb*	*Pb*		*nat1*	*Pb*	*Pb*		*nat1*	*Pb*	*Pb*		*nat1*	*Pb*	*P.b*	
			nat1				nat1				nat1				nat1		
**T**	**Phe**	0	37.5	32.47	**Ser**	0	**33.3**	**19**	**Tyr**	0	28.6	40.7	**Cys**	0	0	43.2	**T**
	**Phe**	100	62.5	67.53	**Ser**	33.3	44.5	21.6	**Tyr**	100	71.4	59.3	**Cys**	100	100	56.8	**C**
	**Leu**	0	0	4.9	**Ser**	0	0	15.2	**Stop**	0	0	31.8	**Stop**	0	0	45.5	**A**
	**Leu**	7.1	0	19.4	**Ser**	44.4	0	14.9	**Stop**	0	0	22.7	**Trp**	100	100	100	**G**
**C**	**Leu**	0	0	19.7	**Pro**	0	30	26.1	**His**	0	25	44.2	**Arg**	0	15.4	18.6	**T**
	**Leu**	38.4	57.1	25.5	**Pro**	40	70	30	**His**	100	75	55.8	**Arg**	38.5	0	29.7	**C**
	**Leu**	0	0	8.4	**Pro**	0	0	25.3	**Gln**	0	0	45.8	**Arg**	0	0	19	**A**
	**Leu**	61.6	42.9	22.1	**Pro**	60	0	18.6	**Gln**	100	100	54.2	**Arg**	61.5	0	13.6	**G**
**A**	**Ile**	0	33.3	36.5	**Thr**	0	0	25.1	**Asn**	0	33,3	40.5	**Ser**	11.15	0	11.7	**T**
	**Ile**	100	66.7	52.7	**Thr**	82.4	70.6	35.7	**Asn**	100	66,7	59.5	**Ser**	11.15	22.3	17.6	**C**
	**Ile**	0	0	10.8	**Thr**	0	0	23.7	**Lys**	0	0	34.1	**Arg**	0	0	11.3	**A**
	**Met**	100	100	100	**Thr**	17.6	29.4	15.5	**Lys**	100	100	65.9	**Arg**	0	0	7.8	**G**
**G**	**Val**	0	33.3	27.2	**Ala**	5.3	31.6	27.5	**Asp**	4.4	47.8	51.8	**Gly**	0	30	29	**T**
	**Val**	73.3	53.3	37.1	**Ala**	47.5	68.4	32.8	**Asp**	95.6	52.2	48.2	**Gly**	45	70	32.7	**C**
	**Val**	0	0	8.6	**Ala**	10.4	0	22	**Glu**	15.4	30.7	42.8	**Gly**	0	0	24.8	**A**
	**Val**	26.7	13.4	27.1	**Ala**	36.8	0	17.7	**Glu**	84.6	69.3	57.2	**Gly**	52.4	0	13.5	**G**

## Supplementary Materials

The following are available online at https://www.mdpi.com/2309-608X/5/4/96/s1, Figure S1: Construction of a *MAT1-2-1* deletion mutant; Figure S2: Construction of a *flbA* deletion strains; Figure S3: Complementation of *MAT1-2-1* and *flbA* deletion strains; Table S1: Codon usage in *P. brevicompactum.* The genomic sequences of strain AgRF18 was used for calculating frequency of preferred amino acid codons; Table S2: Comparison of CAI and GC-content of *nat1* and *Pbnat1*; Table S3: List of oligonucleotides used in this study; Table S4. List of plasmids, used in this study.
