# Supplementary material for: Construction of a Codon-Adapted Nourseotricin-Resistance Marker Gene for Efficient Targeted Gene Deletion in the Mycophenolic Acid Producer Penicillium brevicompactum
Source: J Fungi (Basel). 2019 Oct 10;5(4):96. doi: 10.3390/jof5040096 (PMC6958462; doi:10.3390/jof5040096)
Supplement: Supplementary file 1 [file jof-05-00096-s001.pdf]

Appendix

Table S1. Codon usage in *P. brevicompactum.* The genomic sequences of strain AgRF18 was used for calculating frequency of preferred amino acid codons.

fields: [triplet] [frequency: per thousand] ([number])							
UUU	12.6 (71568)	UCU	16.1 (91342)	UAU	11.3 (64019)	UGU	5.7 (32482)
UUC	26.2 (148291)	UCC	18.3 (103490)	UAC	16.5 (93591)	UGC	7.5 (42450)
UUA	4.4 (24630)	UCA	12.9 (73146)	UAA	0.7 (3697)	UGA	1.0 (5420)
UUG	17.6 (99392)	UCG	12.6 (71116)	UAG	0.5 (3099)	UGG	15.2 (86302)
CUU	17.8 (100530)	CCU	15.6 (88584)	CAU	11.2 (63312)	CGU	11.0 (62065)
CUC	23.1 (130942)	CCC	17.9 (101550)	CAC	13.7 (77695)	CGC	17.5 (99140)
CUA	7.6 (42852)	CCA	15.1 (85606)	CAA	18.6 (105265)	CGA	11.2 (63627)
CUG	20.0 (113406	CCG	11.1 (62688)	CAG	22.0 (124620)	CGG	8.0 (45184)
AUU	18.7 (105906)	ACU	14.8 (83656)	AAU	15.1 (85709)	AGU	9.9 (56091)
AUC	27.0 (152588)	ACC	21.1 (119507)	AAC	22.2 (125473	AGC	14.9 (84326)
AUA	5.5 (31032)	ACA	14.0 (79116)	AAA	15.7 (88997)	AGA	6.7 (38023)
AUG	20.2 (114569)	ACG	9.2 (52330)	AAG	30.3 (171504)	AGG	4.6 (26150)
GUU	16.7 (94447)	GCU	23.1 (130555)	GAU	29.0 (164398)	GGU	19.6 (111162)
GUC	22.8 (128878)	GCC	27.5 (155664)	GAC	27.2 (153773)	GGC	22.1 (124973)
GUA	5.3 (29738)	GCA	18.5 (104642)	GAA	26.0 (146960)	GGA	16.7 (94555)
GUG	16.6 (93739)	GCG	14.8 (83520)	GAG	34.7 (196349)	GGG	9.1 (51732)

Table S2**.** Comparison of CAI and GC-content of *nat1* and *Pbnat1*

Gene	CAI	Total GC content (%)	GC1 (%)	GC2 (%)	GC3 (%)
nat1	0.83	71.2	68.9	48.9	95.8
Pbnat1	0.91	63.7	69.5	48.9	72.6

Table. S3. List of oligonucleotides used in this study

Name	Sequence (5′–3′)	Specificity
5'-Pb-flbA-PstI-for	ATACTGCAGCTCATACAGGCGTCCTCAGCC	5′ flanking region of *flbA*
5′-Pb-flbA-PstI-rv	ATCCTGCAGGAATGGTTTTGAGTCTTCGGGT	5′ flanking region of *flbA*
3'-Pb-flbA-NotI-for	TATTAAGCGGCCGCGATTCCGACTCCTCATGA	3′ flanking region of *flbA*
3'-Pb-flbA-NotI-rv	CATTAGCGGCCGCAGTATCTACCACCTGAGCAACC	3′ flanking region of *flbA*
5′-Pb-MAT1-2-1-MluI-for	ACATAACGCGTCCCTCAACGATGGTCCGCAC	5′ flanking region of M*AT1-2-1*
5'-Pb-MAT1-2-1-EcoRI-rv	ATGTAGAATTCGCACGACGAGGGCTCATGGA	5′ flanking region of *MAT1-2-1*
3'-Pb-MAT1-2-1-NotI-for	ATACTGCGGCCGCGCTTTTCATCCCATCGTTTCT	3′ flanking region of *MAT1-2-1*
3'-Pb-MAT1-2-1-NotI-rv	ATGTAGCGGCCGCGAACCACCAATCATCTCTCT	3′ flanking region of *MAT1-2-1*
Pb-FlbA-5'-genome-for	GGTCGAGCTAAGGGAAGATA	5′ flanking region of *flbA*
Pb-FlbA-3'-genome-rv	CGCATGCTTTGGCCACAAGA	3′ flanking region of *flbA*
Pb_flbA-for	CCAACTCAACCCGGAAACCA	*flbA*
Pb-flbA-rv	GCTCATGCTGCGTTCAGGGAT	*flbA*
4736-f	ACTTTCATCTGGGCCAGCGAGTGG	*apn2*
2756-r	GCCCGCCAGCGTCTGGGCGAAATG	*sla2*
Pb_spec_MAT1-2-1-f	CCTGGAGTTACCACCTACTC	*MAT1-2-1*
Pb_MAT1-2_r	TGATGTCCATGTAGTCGGTC	*MAT1-2-1*
PtrpC_seq_r	CTCCACTAGCTCCAGCCAAG	*ptrpC*
Pbnat1-s	CTCGATGACACGGCTTACCGCTA	*Pbnat1*
Tn5-phleo-BoxI-for	GAATAGACTTACGTCCATGGGCGAAATGACCGACC	*ble*
Tn5-phleo-ApaI-rv	GAATTGGGCCCTCATGAGATGCCTGCAAGCA	*ble*
MAT1-2-1-OE-EcoRI-for	GATTATGAATTCGAGCCCTCGTCGTGCCATG	*MAT1-2-1*

MAT1-2-1_EcoRI-OE-rv	GGCGCGGAATTCGGACATTGAGAC TGAAGGCAG	*MAT1-2-1*
Pb-flbA-BglII-OE-for	GCGGCTAGATCTATGCCAACTCAAC CCGGAAA	*flbA*
Pb-flbA-BamHI-OE-rv	GATTATGGATCCTTGTCAGGCGCGG GCTGAAC	*flbA*

Table S4. List of plasmids, used in this study

**Name**	**Characteristics**	**source**
pDrive/ptpc-Tn5Phleo	*trpC* promoter of *Aspergillus nidulans, ble* resistance gene of *Streptoalloteichus hindustanus*	Böhm et al., 2013
P17831-nat1	*gpd* promoter of *A.nidulans*, *egfp*, *TtrpC* of *A. nidulans*, *trpC* promoter of *A. nidulans*, *nat1* resistance gene of *S. noursei*	Gesing et al. 2012
PN-EGFP	*gpd* promoter of *A.nidulans*, *egfp*, *TtrpC* of *A. nidulans*, *trpC* promoter of *A. nidulans, hph* resistance gene of *Streptomyces hygroscopicus*	Kück et al., 2009
pPtrpC-nat1	*trpC* promoter of *A. nidulans, nat1* resistance gene of *S. noursei,*	Kück et al. 2009
pPtrpC-Pbnat1	*trpC* promoter of *A. nidulans,* codon adapted synthetized *Pbnat1* resistance gene	This study
pPb-MAT1-2-1-KO	5′ flanking region of *MAT1-2-1* gene, *trpC* promoter of *A. nidulans*, *Pbnat1* (codon adapted *nat1* resistance gene of *S. noursei*), 3′ flanking region of *MAT1-2-1* gene	This study
pPb-flbA-KO	5′ flanking region of *flbA* gene, *trpC* promoter of *A. nidulans, Pbnat1*(codon adapted *nat1* resistance gene from *S. noursei*), 3′ flanking region of *flbA* gene	This study
pPb-MAT1-2-1-comp	*gpd* promoter of *A.nidulans*, *egfp*, *MAT1-2-1* gene from *P. brevicompactum TtrpC* of *A. nidulans*	This study
pPb-flbA-comp	*gpd* promoter of *A.nidulans*, *egfp*, *flbA1* gene from *P. brevicompactum TtrpC* of *A. nidulans*	This study

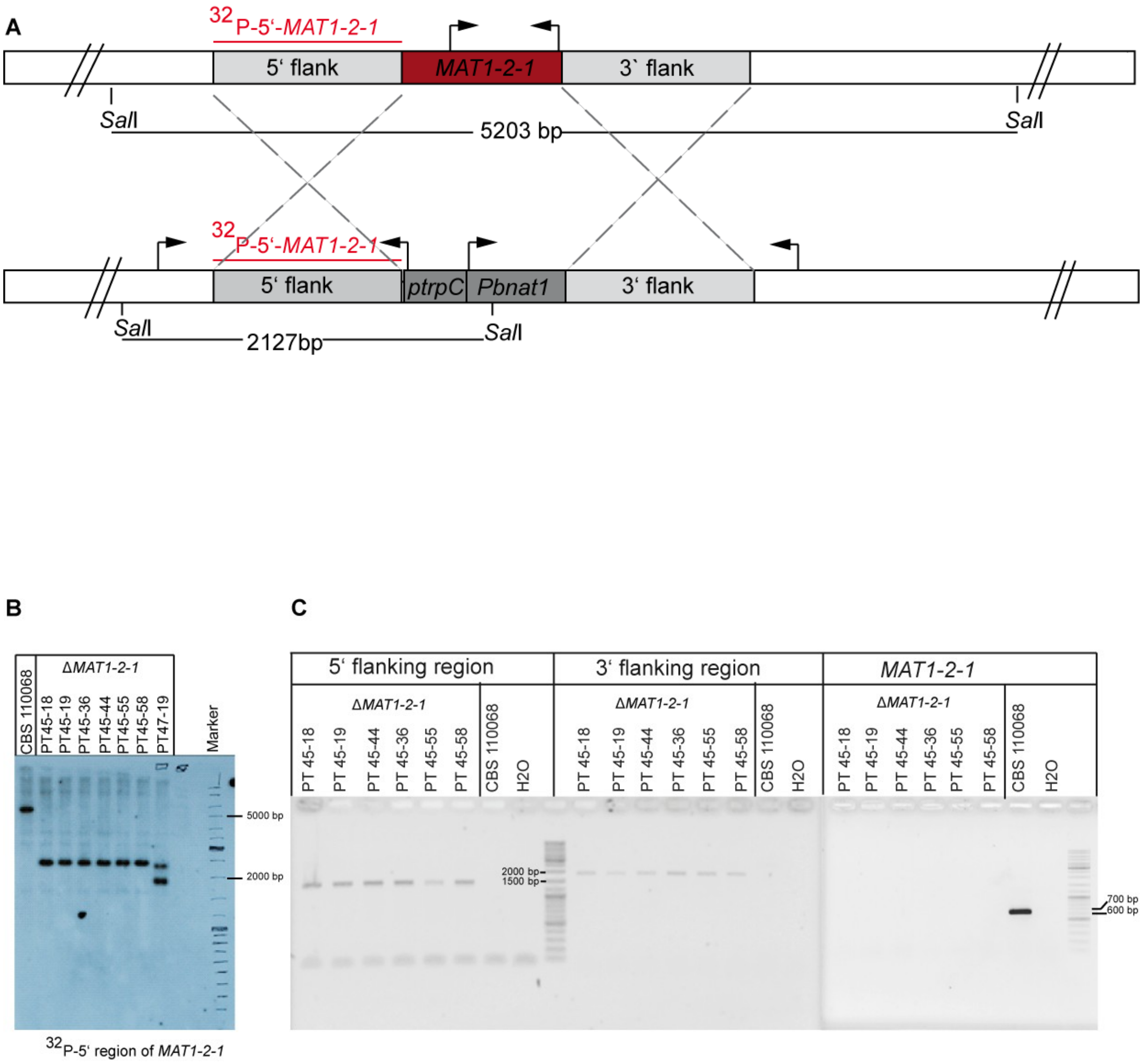

Figure S1.: Construction of a *MAT1-2-1* deletion mutant. (A) *MAT1-2-1* locus in the reference strain and in the deletion mutant Δ*MAT1-2-1.* Dashed lines indicated the homologous recombination event. Arrows show the primer pairs used for PCR analyis. Restriction enzyme recognition site used for digestion of genomic DNA and the size of corresponding fragments are indicated. (B) Autoradiograph of a Southern hybridization analysis. 5′ flanking regions served as radioactive labelled probes (C) Verification of recombinant strains with PCR. Numbers above autoradiogram and gels indicate individual transformants.

A

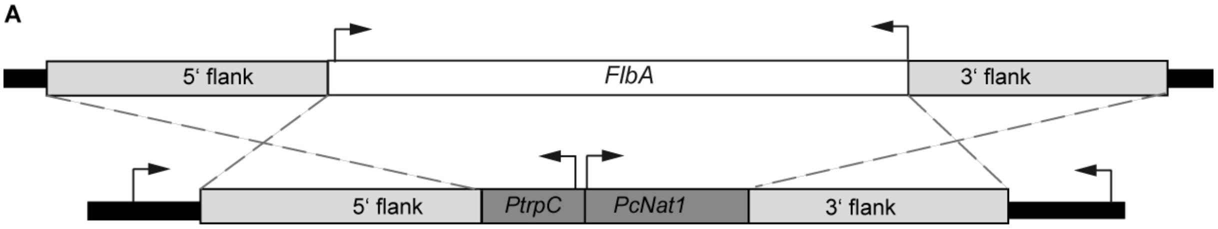

B

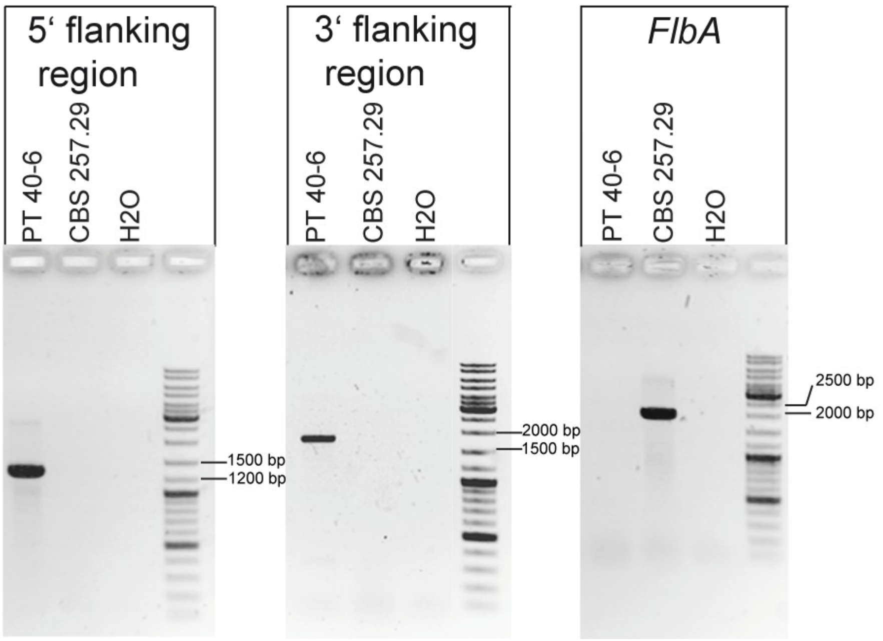

Figure S2. Construction of a *flbA* deletion strains. (A*) flbA* locus in the reference strain and in the deletion mutant Δ*flbA.* Dashed lines indicated the homologous recombination event (B) Verification of recombinant strains with PCR. The arrows shown in (A) represent the primer pairs used for PCR analysis. CBS 257.29: wild type; PT 40-6: Δ*flbA*.

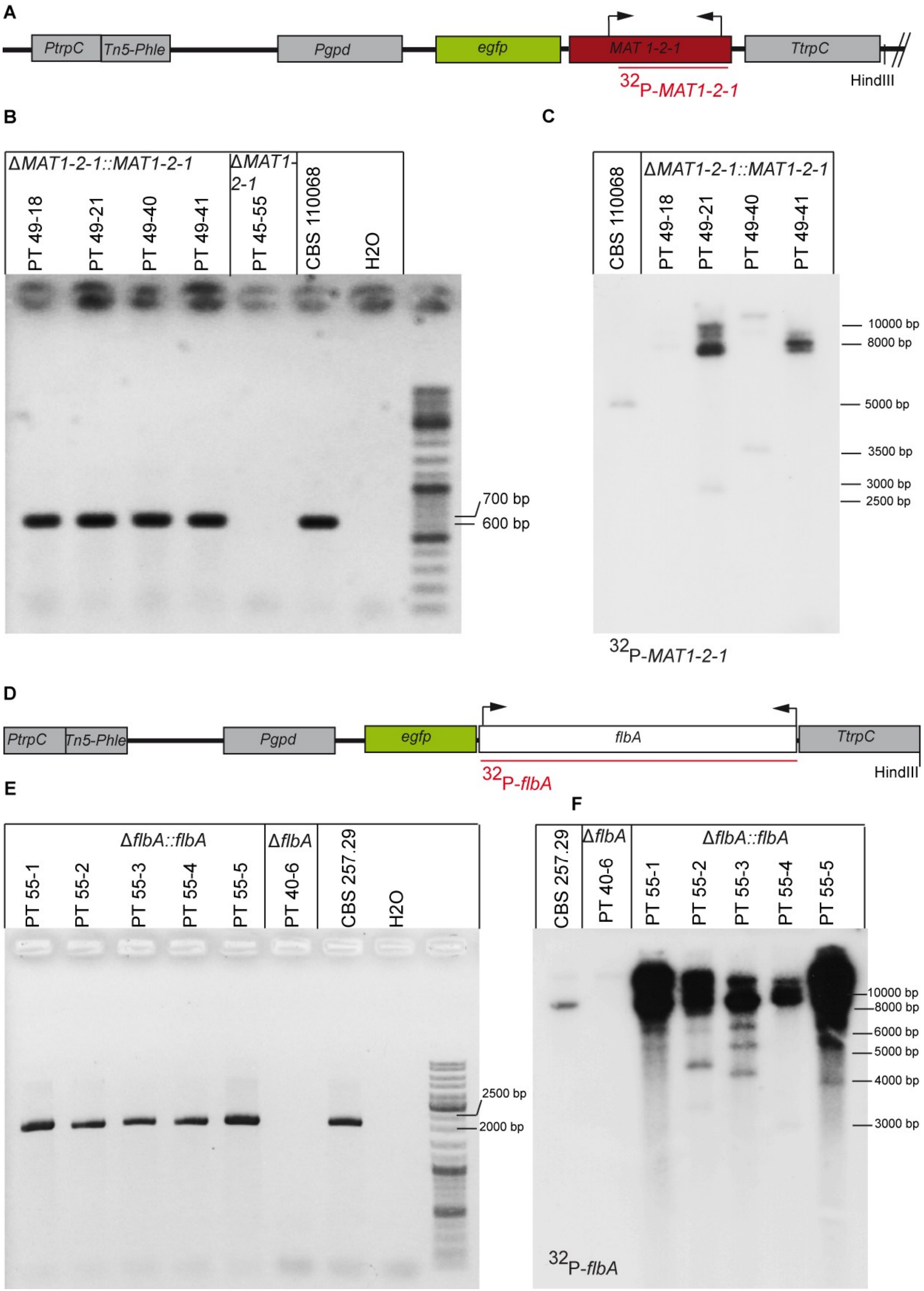

Figure S3. Complementation of *MAT1-2-1 and flbA* deletion strains. (A) Vector map for integration of the *MAT1-2-1* gene into the corresponding deletion strains. (B) PCR analysis for verification of *MAT1-2-1* recombinant strains (C) Evidence for complementation of Δ*MAT1-2-1* by Southern hybridization

with radioactively labeled probe specific for the *MAT1-2-1* gene. (D) Schematic map of construct used for complementation of *flbA* deletion strains. (E) PCR analysis to prove the genomic integration of *flbA* gene in deletion strains. CBS 257.29 & CBS 110068 are wild-type strains. (F) Evidence for complementation of Δ*flbA* by Southern hybridization with radioactively labeled probe specific for the *flbA* gene. Arrows in A & D show the position of primer pairs used for PCR.
